# Geospatial Patterning of Hypertension and High Cholesterol Prevalence in Inland Southern California: An Ecological ZIP-Code-Level Study

**DOI:** 10.7759/cureus.107860

**Published:** 2026-04-28

**Authors:** Anton Andricioaei, Andrea Bogdan, Emma Dutt, Sodongoo Sodtuya, Arvin Motamedi Azari

**Affiliations:** 1 Department of Anthropology, University of California Riverside, Riverside, USA; 2 Department of Psychology, University of California Riverside, Riverside, USA; 3 Department of Biology, University of California Riverside, Riverside, USA; 4 Department of Political Science, University High School, Irvine, USA; 5 Department of Nursing, San Diego State University, San Diego, USA

**Keywords:** cardiovascular risk, geospatial analysis, healthcare disparities, high cholesterol, hypertension, inland empire, obesity, preventive care, socioeconomic factors, spatial epidemiology

## Abstract

Background

California’s Inland Empire (IE) is a federally designated Health Professional Shortage Area (HPSA) with high burdens of cardiovascular disease (CVD). Given limited regional health surveillance, this study assesses spatial clustering of hypertension and high cholesterol and associations between these outcomes and neighborhood-level demographic, socioeconomic, and health characteristics in spatially adjusted models.

Methods

This cross-sectional ecological study uses data from the 2025 CDC Population Level Analysis and Community Estimates (PLACES) dataset and 2019-2023 American Community Survey (ACS), representing 132 ZIP Code Tabulation Areas (ZCTAs) in Riverside and San Bernardino counties. With respect to data analysis, a spatial econometric workflow, variance inflation factors (VIF), diagnostic ordinary least squares (OLS) regression, Moran’s I for geospatial clustering, and spatial error models (SEM) were applied. All results in this study are presented as hypothesis-generating.

Results

The mean modeled prevalence across ZCTAs was 34.3% for hypertension and 36.5% for high cholesterol, with significant geospatial clustering being present for hypertension and cholesterol (I = 0.293, p < 0.001; I = 0.162, p = 0.002). Additionally, in SEM-adjusted models, both outcomes were associated with obesity (β = 0.584, p < 0.001; β = 0.308, p < 0.001) and recent checkups (β = 1.395, p < 0.001; β = 1.215, p < 0.001), and negatively associated with median income (β = −0.465, p < 0.001; β = −0.180, p < 0.001).

Conclusions

Modeled hypertension and high cholesterol prevalence in the IE varied by ZCTA and were spatially clustered, while obesity, income, and healthcare engagement were associated with neighborhood-level cardiovascular risk. These results support the use of spatial surveillance and may inform future public health research and practice in these medically underserved regions.

## Introduction

In the United States, high cholesterol and hypertension are prevalent modifiable risk factors for cardiovascular disease (CVD), not limited to stroke, atherosclerosis, hypertensive heart disease, and heart failure [1,2]. According to the National Health and Nutrition Examination Survey (NHANES), nearly half of adults aged 18 or older have hypertension, while approximately 11.4% of adults aged 20 or older have high total cholesterol levels [3,4]. Although these conditions are largely preventable through lifestyle changes (e.g., regular exercise and limiting saturated fat intake) and are treatable with appropriate therapies (e.g., statins for hypercholesterolemia and antihypertensive medications), their prevalence varies across socioeconomic, racial, and geographic groups [5,6]. Overall, hypertension and higher cholesterol prevalence tend to be high among African American populations, individuals with obesity, and in low-income communities [1,5-7]. Moreover, communities with limited access to routine screening and medical resources often face barriers to care, which may contribute to disparities in diagnosis and treatment, particularly among certain Hispanic communities [6].

Within this national context, California tends to have lower rates of cardiovascular risk factors and CVD, yet notable disparities are present between high- and low-income counties [8]. Moreover, the Inland Empire (IE) region of Southern California consists of Riverside and San Bernardino Counties and is home to a predominantly Hispanic population, faces high levels of poverty, has a relatively young age structure, and is experiencing rapid population growth [9]. Additionally, nearly one-third of residents report living in Health Professional Shortage Areas (HPSA), indicating that there is insufficient primary care provider coverage in certain parts of the region [10]. In this environment, Riverside County has recently experienced an increase in the mortality rate for hypertensive heart disease, rising from 31.3 per 100,000 residents in 2018 to 35.1 per 100,000 residents in 2022, while the mortality rate for African-American adults is double that of White adults [11]. Additionally, between 2022 and 2024, San Bernardino County saw a nearly 16% increase in hospitalization due to heart failure, from an incidence of 402.2 per 100,000 residents in 2022 to 467.7 per 100,000 residents in 2024, while the state saw a decrease from 380.7 per 100,000 to 363.9 per 100,000 residents. Also, in 2024, the hospitalization due to hypertension (82.3 per 100,000 residents) was greater than that of the state average (56.2 per 100,000 residents) [12]. Given these disparities, recent increases in chronic disease, and ongoing preventive care shortages, understanding how cardiovascular risk differs at the neighborhood level is important for addressing the region’s unmet medical needs.

Furthermore, surveillance efforts tend to operate at larger state or regional scales, which can mask variation in cardiovascular risk that may otherwise be observed at smaller geographic areas, such as counties [13]. Similarly, county-level surveillance may experience specificity issues, while ZIP Code Tabulation Area (ZCTA)-level modeling allows for more granular representations of geospatial patterns relating to built environments, healthcare accessibility, socioeconomic data, and chronic disease prevalence [14]. Specifically, these factors can vary within counties and influence cardiovascular risk through mechanisms not limited to access to preventive care, food environments, and opportunities for physical activity [6]. Meanwhile, in the IE, descriptive regional assessments conducted at the postal ZIP code level may not apply appropriate spatial analyses to identify geographic disparities [15]. Although these reports have identified broader racial disparities through mixed-method surveys, they reflect populations distributed across both counties and may not accurately capture neighborhood-level differences in socioeconomic status, built environment, and healthcare access [11,16].

Therefore, the objective of this study was to examine ZCTA-level variation in modeled hypertension and high cholesterol prevalence across the IE, specifically to characterize spatial clustering using Moran’s I, and assess associations between neighborhood-level characteristics and these outcomes using spatially adjusted models (SEM). Using CDC Population Level Analysis and Community Estimates (PLACES) modeled estimates and demographic data and socioeconomic data from the American Community Survey (ACS), this analysis evaluates relationships between cardiovascular risk factors and neighborhood-level characteristics, including race, obesity prevalence, median household income, and recent engagement with preventive care services [17,18]. In this study of a medically underserved area, a spatial econometric workflow was used to produce hypothesis-generating results that may provide areas for future public health investigation.

## Materials and methods

The study was conducted at the University of California, Riverside (Riverside, CA, USA). This study employed a cross-sectional ecological design to assess associations between neighborhood-level (ZCTA) characteristics and hypertension and high cholesterol prevalence across 132 ZCTAs in Riverside and San Bernardino counties, while spatial clustering of these outcomes was also examined. Specifically, each ZCTA represented a neighborhood that had health, demographic, social, and economic characteristics assigned to it.

In order to obtain health-related variables at the ZCTA-level, the 2025 CDC PLACES Geographic Information System (GIS)-Friendly dataset was used to obtain the crude prevalence of high blood pressure (BPHIGH_CrudePrev) and high cholesterol (HIGHCHOL_CrudePrev) measured by the percentage of adults aged 18 and older who reported ever being told by a healthcare provider that they had the respective risk factor. It is important to note that these measures reflect lifetime self-reported diagnoses rather than extant clinical prevalence. These estimates were derived from the 2023 Behavioral Risk Factor Surveillance System (BRFSS) and benchmarked to the 2020 U.S. Census using the CDC’s Small Area Estimation (SAE) technique [17,19]. For reference, BRFSS is the world’s largest health-related telephone survey, which collects data on behavior, chronic health conditions, and preventive care use and conducts close to 400,000 health interviews annually [20]. Specifically, self-reported high blood pressure and high cholesterol served as proxies for hypertension and high cholesterol prevalence, respectively. Furthermore, this dataset provided predictor variables such as estimates for the percentage of adults who reported being obese (BMI ≥ 30) and the percentage of adults who had a routine medical checkup within the past year [17].

Given the study's emphasis on race/ethnicity and socioeconomic status, 2019-2023 ACS Five-Year Table Counts were used to provide ZCTA-level total population counts (B01003), Black (B02001) population, and Hispanic (B03003) population counts for Riverside and San Bernardino Counties. ACS also provided median household income (B19013) data for each ZCTA [18]. After exporting ACS table values to Excel, sociodemographic and economic indicators were merged with the previously mentioned health-related predictors and outcomes using ZCTA identifiers to create a single analytic dataset, where each row represented one ZCTA in the IE. Within R, the proportions for Black and Hispanic residents were calculated by dividing subgroup counts by the total ZCTA population and multiplying by 100 to generate percentages, and median income was scaled per $10,000. Following data processing, observations with missing values across study variables were removed using na.omit(). Moreover, the ACS dataset included 164 ZCTAs in the IE, whereas complete CDC PLACES data were available for 132 ZCTAs. Thus, ZCTAs without complete overlap between ACS and CDC PLACES data (n = 32), primarily due to missing or suppressed estimates in the CDC PLACES dataset and/or unmatched ACS records, were excluded, resulting in a final analytic sample of 132 ZCTAs (N = 132).

Following this process, descriptive statistics, including means, standard deviations, medians, skewness, and ranges, were calculated and summarized in a descriptive statistics table. Given that regression models were applied, multicollinearity was assessed using variance inflation factors (VIFs), with values below five used as a general guideline for low multicollinearity. This cutoff was based on literature suggesting that values greater than five indicate problematic levels of multicollinearity [21]. An initial VIF test was then conducted in which the percentage of residents aged ≥65 years (sourced from ACS) was evaluated alongside the other predictors: % Black residents, % Hispanic residents, % obese, median income (per $10,000), and % recent checkup. Since substantial multicollinearity and model instability were observed between age ≥65 and recent checkup prevalence (VIF = 8.48 and 11.68, respectively) when both variables were included, likely reflecting overlap in healthcare utilization patterns, recent checkup prevalence was retained as it more directly reflects healthcare engagement and the likelihood of diagnosis, and age ≥65 was excluded [22]. Although age remains a key determinant of cardiovascular risk, this decision may introduce residual confounding, which is further discussed in the limitations section [1]. After age was omitted, VIF testing was repeated for the remaining variables, all of which were below the specified threshold, and these values are reported in the Results section.

After VIF testing, we performed two diagnostic ordinary least squares (OLS) models, one for each outcome, and applied heteroskedasticity-robust standard errors (HC1 estimator) to ensure that any potential heteroskedasticity was accounted for. Both OLS and spatial models were specified as: outcome ~ % Black residents + % Hispanic residents + % Obese + median income (per $10,000) + % recent checkup. Within these models, we also calculated R and adjusted R² to understand how well these models explained outcome variability in a linear model. Notably, the values for R and adjusted R² were high, which may be due to data characteristics found within CDC PLACES modeled estimates. Mathematically, OLS models assume that residuals are spatially independent, yet if statistically significant spatial autocorrelation is present, the assumption is violated, warranting the use of spatial models to correct for that misspecification [23]. Therefore, Moran’s I statistics were calculated to test for spatial autocorrelation, with statistical significance set at p < 0.05 [24].

Given the evidence of spatial autocorrelation for both outcomes, spatial error models (SEMs) were fitted to account for spatially correlated error in the regression models. Specifically, SEMs were applied over spatial lag models (SLMs) because the study does not aim to model diffusion of outcomes between ZCTAs but rather to compare ZCTAs while accounting for spatial dependence in the error structure that may bias these relationships [23]. In order to perform the SEM analysis, a row-standardized queen’s first-order contiguity spatial weights matrix was constructed using 2020 U.S. Census Topologically Integrated Geographic Encoding and Referencing (TIGER)/Line ZCTA shapefiles. Specifically, neighbors were defined as ZCTAs sharing borders or vertices (style = “W”), as this approach is well-suited for irregularly shaped areas and ensures that adjacent spatial relationships are captured. ZCTAs without complete data were excluded prior to spatial weight construction, while seven ZCTAs without neighbors were retained using a zero-policy approach (zero.policy = TRUE). The outputs of the SEMs were spatially adjusted beta coefficients, 95% confidence intervals, p-values, and spatial error coefficients (λ). In order to ensure that spatial dependence was fully addressed, Moran’s I was recalculated on SEM residuals. Finally, spatial patterns in outcome prevalence were mapped using choropleth maps generated with the ggplot2 and sf packages in R. These maps used ZCTA and county boundaries from the 2020 TIGER/Line shapefiles, with a consistent color scale applied across both maps to ensure comparability and readability.

All analyses were conducted using R version 4.4.2 (R Foundation for Statistical Computing, Vienna, Austria). The following packages were used: dplyr, ggplot2, sf, psych, car, lmtest, broom, and spdep. Key functions included lm() for OLS estimation, vcovHC() for robust standard errors, vif() for multicollinearity diagnostics, moran.test() for spatial autocorrelation, poly2nb() and nb2listw() for spatial weights construction, and errorsarlm() for SEM estimation.

## Results

Descriptive statistics

In Table 1, the descriptive statistics for the 132 ZCTAs analyzed in this study are listed. Across the region, the mean modeled prevalence of hypertension was 34.27%, while the mean prevalence for high cholesterol was 36.53%. With respect to demographic composition, IE ZCTAs had an average of 6.31% Black residents and 46.48% Hispanic residents. Finally, the average median household income was $84,800, which was scaled per $10,000.

**Table 1:** Descriptive statistics for cardiovascular risk outcomes and predictors in Inland Southern California (N = 132) Hypertension (%) = percentage of adults (18+) with self-reported diagnosed high blood pressure; high cholesterol (%) = percentage of adults (18+) with self-reported diagnosed high cholesterol; % obese = percentage of adults (18+) with BMI ≥30; % Black = percentage of population identifying as Black residents; % Hispanic = percentage of population identifying as Hispanic residents; % recent checkup = percentage of adults (18+) who had a checkup <1 year. Data sources: 2025 CDC Population Level Analysis and Community Estimates (PLACES); 2019-2023 American Community Survey (ACS).

Predictor	Mean	Standard Deviation	Median	Min	Max	Range	Skew	Kurtosis	Standard Error
Hypertension (%)	34.27	5.1	32.9	16.5	49.5	33.00	0.51	0.85	0.44
High Cholesterol (%)	36.53	4.08	35.8	18.6	50.4	31.8	0.26	3.04	0.36
% Black	6.31	4.72	5.09	0.01	24.53	24.52	1.23	1.76	0.41
% Hispanic	46.48	19.42	45.92	4.82	96.76	91.94	0.18	-0.76	1.69
% Obese	35.15	3.78	35.25	24.9	44	19.1	-0.18	-0.3	0.33
Median Income (per $10,000)	8.48	2.52	8.26	3.61	15.71	12.1	0.51	-0.09	0.22
% Recent Checkup	71.69	3.57	71.4	60.4	85.5	25.1	0.74	2.09	0.31

Multicollinearity diagnostics

In Table 2, we report the multicollinearity diagnostics, which were measured by VIF. For both models, low collinearity (VIF < 5) was observed among all predictors: % Black residents (VIF=1.22), % Hispanic residents (VIF = 2.98), % obese (VIF = 2.12), median income (VIF = 1.49), % recent check up (VIF = 2.50).

**Table 2:** Variance inflation factors for modeled hypertension and high cholesterol prevalence models in Inland Southern California (N = 132) All variables are defined in Table 1. Data sources: 2025 CDC Population Level Analysis and Community Estimates (PLACES); 2019-2023 American Community Survey (ACS).

Predictor	Hypertension	High Cholesterol
% Black	1.22	1.22
% Hispanic	2.98	2.98
% Adults With Obesity	2.12	2.12
Median Income	1.49	1.49
% Recent Check Up	2.50	2.50

OLS regression

In Table 3, the diagnostic OLS regression model for hypertension is outlined. In this model, positive associations were present between the percentage of residents reporting a recent checkup and obesity prevalence. Furthermore, median household income was negatively associated with hypertension prevalence, while proportions of Black and Hispanic residents were found to be statistically insignificant.

**Table 3:** Ordinary least squares (OLS) regression results for modeled hypertension prevalence in Inland Southern California (N = 132) Around 89.8% of the variance (R² = 0.898; adjusted R² = 0.894). All variables are defined in Table 1. Data sources: 2025 CDC Population Level Analysis and Community Estimates (PLACES); 2019-2023 American Community Survey (ACS).

Term	β-coefficient	Standard Error	95% CI	p-value
Intercept	-64.00	4.838	-73.57 to -54.42	<0.001
% Black	0.022	0.034	-0.045 to 0.089	0.521
% Hispanic	0.010	0.013	-0.015 to 0.036	0.430
% Obesity	0.261	0.056	0.150 to 0.371	<0.001
Median Income	-0.875	0.070	-1.014 to -0.735	<0.001
% Recent Checkup	1.338	0.064	1.210 to 1.465	<0.001

Furthermore, Table 4 contains the diagnostic high cholesterol OLS model, where negative associations were observed for the percentage of Black residents and median income. Also, positive associations were noted for the percentage of Hispanic residents, obese adults, and adults who received a recent checkup. 

**Table 4:** Ordinary least squares (OLS) regression results for modeled high cholesterol prevalence in Inland Southern California (N = 132) R² = 0.953; adjusted R² = 0.951. All variables are defined in Table 1. Data sources: 2025 CDC Population Level Analysis and Community Estimates (PLACES); 2019-2023 American Community Survey (ACS).

Term	β-coefficient	Standard Error	95% CI	p-value
Intercept	-53.69	2.623	-58.88 to -48.50	<0.001
% Black	-0.118	0.018	-0.155 to -0.082	<0.001
% Hispanic	0.034	0.007	0.020 to 0.048	<0.001
% Obesity	0.188	0.030	0.128 to 0.247	<0.001
Median Income	-0.297	0.038	-0.372 to -0.221	<0.001
% Recent Checkup	1.190	0.035	1.121 to 1.259	<0.001

SEM

Moreover, Moran’s I testing of hypertension OLS residuals indicated that significant spatial autocorrelation (I = 0.293, p < 0.001) was present, thereby showing that the outcomes were spatially dependent and geographically clustered. With the SEM applied, obesity (β = 0.584, p < 0.001) and recent checkup prevalence (β = 1.395, p < 0.001) were positively associated with hypertension, while median income was negatively associated (β = −0.465, p < 0.001). Within this updated model, the percentage of Black residents was negatively associated and statistically significant (β = −0.054, p = 0.048), which was in contrast to Hispanic population percentages, where the relationship became non-significant (p = 0.856). The spatial error parameter was statistically significant (λ = 0.786, p < 0.001), confirming that spatially structured residual variation was present. These results are outlined in Table 5, with corresponding coefficients, standard errors, confidence intervals, and p-values.

**Table 5:** Spatial error model (SEM) results for modeled hypertension prevalence in Inland Southern California (N = 132) All variables are defined in Table 1. Data sources: 2025 CDC Population Level Analysis and Community Estimates (PLACES); 2019-2023 American Community Survey (ACS).

Term	β-coefficient	Standard Error	95% CI	p-value
Intercept	-81.67	4.356	-90.212 to -73.137	<0.001
% Black	-0.054	0.027	-0.108 to -0.001	0.048
% Hispanic	-0.002	0.013	-0.027 to 0.022	0.856
% Obesity	0.584	0.058	0.471 to 0.697	<0.001
Median Income	-0.465	0.062	-0.587 to -0.343	<0.001
% Recent Checkup	1.395	0.056	1.284 to 1.506	<0.001
Spatial Error (λ)	0.786	0.056	0.676 to 0.896	<0.001

Similar to the hypertension model, Moran’s I testing specified that spatial clustering was present in the OLS residuals for high cholesterol (I = 0.162, p = 0.002). Within the SEM model, associations for both Black population percentage (β = −0.130, p < 0.001) and income (β = −0.180, p < 0.001) were negative. Also, positive associations were present for the percentage of Hispanic populations (β = 0.026, p < 0.001), obesity (β = 0.308, p < 0.001), and recent checkup prevalence (β = 1.215, p < 0.001). As in the hypertension model, the spatial error parameter was also statistically significant, yet the magnitude was smaller (λ = 0.630, p < 0.001). These results are visible in Table 6. 

**Table 6:** Spatial error model (SEM) results for modeled high cholesterol in inland Southern California (N = 132) All variables are defined in Table 1. Data sources: 2025 CDC Population Level Analysis and Community Estimates (PLACES); 2019-2023 American Community Survey (ACS).

Term	β-coefficient	Standard Error	95% CI	p-value
Intercept	-60.39	2.772	-65.83 to -54.96	<0.001
% Black	-0.130	0.018	-0.165 to -0.094	<0.001
% Hispanic	0.026	0.008	0.010 to 0.042	<0.001
% Obesity	0.308	0.036	0.238 to 0.378	<0.001
Median Income	-0.180	0.040	-0.258 to -0.102	<0.001
% Recent Checkup	1.215	0.035	1.146 to 1.284	<0.001
Spatial Error (λ)	0.630	0.080	0.473 to 0.787	<0.001

Descriptive mapping and residual checks

**Figure 1:**
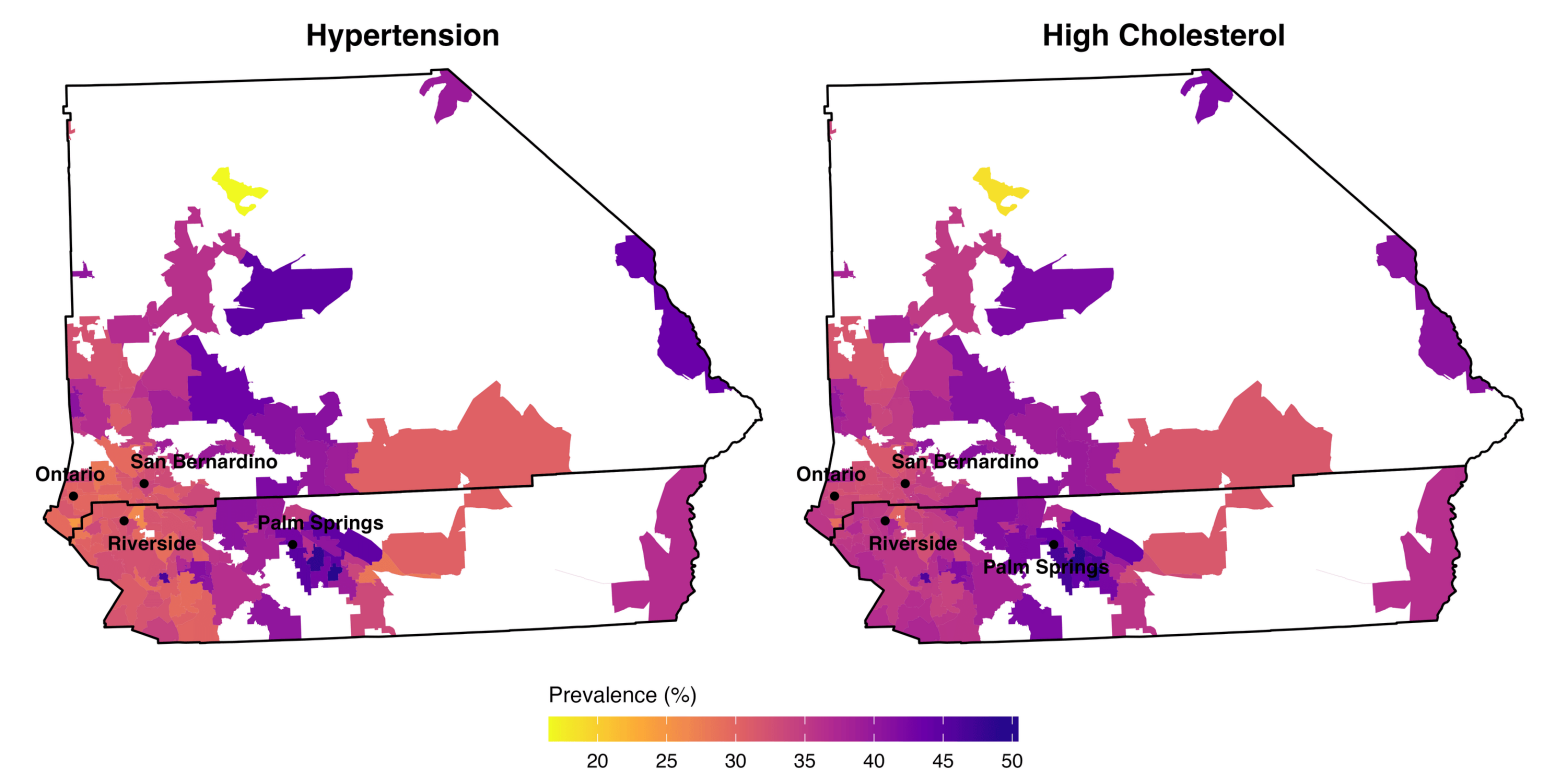
Modeled prevalence of hypertension and high cholesterol across 132 ZIP Code Tabulation Areas in Inland Southern California (N = 132) Hypertension = percentage of adults (≥18 years) with self-reported diagnosis of high blood pressure; high cholesterol = percentage of adults (≥18 years) with self-reported diagnosis of high cholesterol; San Bernardino County (top) and Riverside County (bottom); major urban centers shown include Riverside, San Bernardino, Ontario, and Palm Springs. Data sources: 2025 CDC Population Level Analysis and Community Estimates (PLACES); 2019-2023 American Community Survey (ACS). The figure was created using R 4.4.2 (R Foundation for Statistical Computing, Vienna, Austria) (ggplot2 and sf packages) using data from CDC PLACES 2025 and 2020 U.S. Census Bureau TIGER/LINE shapefiles. The TIGER/Line shapefiles are available at: https://www.census.gov/geographies/mapping-files/time-series/geo/tiger-line-file.2020.html

Finally, Figure 1 represents the descriptive choropleths for both outcomes. Specifically, denser concentrations of hypertension and high cholesterol were visible in southeastern ZCTAs, while high cholesterol appeared to be more evenly distributed. Following this, post-estimation Moran’s I testing of SEM residuals confirmed that spatial autocorrelation had been effectively addressed for both hypertension (I = −0.07, p = 0.874) and cholesterol (I = 0.008, p = 0.394).

## Discussion

Within Riverside and San Bernardino, substantial differences in modeled ZCTA-level hypertension and high cholesterol were present across the region. Although OLS models demonstrated associations between neighborhood-level demographic, socioeconomic, and health-related characteristics and these cardiovascular risk factors, significant spatial autocorrelation in residuals indicated that the geographic independence assumption was violated. After applying SEM, residual spatial dependence was reduced to non-significant levels, suggesting that unmeasured, spatially clustered neighborhood-level factors may influence the distribution of these risk factors in the IE. While spatial clustering findings are directly supported by the data, these patterns are descriptive in nature, and explanatory interpretations of associations between neighborhood-level characteristics and cardiovascular outcomes should be considered hypothesis-generating, given the ecological and cross-sectional design.

The observed strong positive association between obesity and cardiovascular risk (hypertension and high cholesterol) is consistent with the established body of literature connecting high body mass index to cardiovascular risk [25]. Within the context of the IE, obesity is frequently noted as a key concern for residents, stakeholders, and public health departments across both counties [11,16]. These associations suggest that further research may explore obesity-related risk factors and related influences such as diet, physical activity, and healthcare access in high-risk IE communities. Furthermore, the inverse association between income and cardiovascular risk aligns with national health disparities research, in which higher socioeconomic status has been associated with lower prevalence of these risk factors. This pattern may reflect differences in healthcare access, health literacy, and built environments that support long-term healthy behaviors [6]. These findings support further investigation into whether low median household income reflects broader patterns of need across neighborhoods in the IE. Moreover, the positive association between regular checkups and both outcomes may be related to increased detection, given that more frequent interactions with the healthcare system increase the likelihood of diagnosis [26]. Accordingly, this pattern may suggest differences in risk-factor detection across communities and may support further investigation into screening access and utilization at the population level.

In assessing racial disparities in the IE, race and ethnicity associations should be interpreted carefully to avoid biological determinism. Existing studies indicate that disparities in cardiovascular risk among Black and Hispanic populations are largely shaped by structural barriers to care rather than inherent biological differences [27]. In this context, the observed negative association between the percentage of Black residents and hypertension may reflect differences in hypertension awareness, diagnosis, or other unmeasured structural and spatial factors rather than true differences in underlying risk, although these mechanisms cannot be directly evaluated in the present study [28]. Additionally, the geographic dispersion of Black populations across ZCTAs with differing socioeconomic and environmental characteristics may contribute to variability in these associations, and the shift from a non-significant association in OLS to a significant negative association in the SEM likely reflects spatial confounding rather than a true suppressor effect. Thus, accounting for spatially correlated error may be capturing unmeasured neighborhood-level factors that were not represented in the initial model. In contrast, the positive association between greater neighborhood Hispanic composition and high cholesterol may reflect differences in preventive screening utilization and access to care across racial and ethnic groups [29]. The lack of a significant association between Hispanic population percentage and hypertension in the SEM, alongside a positive association for high cholesterol, suggests that these relationships may be outcome-specific and influenced by differences in detection and screening practices. In particular, blood pressure is more routinely assessed in clinical settings, whereas lipid screening may vary more across communities, potentially contributing to this divergence [27]. These findings underscore the need for further research using data that precisely capture healthcare access, screening utilization, and neighborhood-level characteristics, such as healthcare availability and built environment factors, to better understand how cardiovascular risk and detection vary across communities.

Finally, the presence of geospatial clustering and the large magnitude of spatial error in both models suggests that substantial residual variation may be driven by unmeasured, spatially structured factors, such as built environment characteristics, food access, healthcare availability, and transportation infrastructure, which have been associated with cardiovascular risk [6]. Thus, these factors may serve as candidate variables in future research studies examining the region’s unmet medical needs and high CVD burden. Moreover, the most recent IE community health assessment conducted by Loma Linda University has outlined these factors as regional deficiencies that may contribute to poor health outcomes across the region [30]. Subregionally, the descriptive choropleths indicate that areas within the Coachella Valley tend to have higher rates for both risk factors, which is consistent with literature highlighting barriers to care and health disparities among disadvantaged Hispanic farmworker populations [31]. Although trends in this study are descriptive and do not establish underlying mechanisms, the observed spatial patterning (including mapping and Moran’s I) and large magnitude of spatial error support the value of geographically informed surveillance for cardiovascular risk factors in the IE. Moreover, these patterns support the consideration of place-based strategies, such as community-level screening programs, improved access to preventive care services, and efforts to address structural barriers to care.

This study has several limitations, which ought to be addressed accordingly. Primarily, the analysis performed in this study was based on self-reported data, which introduces the risk of recall and desirability bias. Since self-report data serves as a proxy for both outcomes, generalizability may also be of concern, as many other studies use clinical definitions for hypertension (e.g., 130/80 mmHg or lipid panel counts). Additionally, this study follows an ecological and cross-sectional design; therefore, causality cannot be determined, and results may be subject to the ecological fallacy, as population-level associations may not reflect individual-level relationships. Furthermore, the use of CDC PLACES modeled estimates may influence associations and model fit, as these estimates are derived using SAE techniques that borrow strength from covariates, potentially introducing correlation between predictors and outcomes. This may contribute to inflated model fit statistics (e.g., R and R²), which should be interpreted with caution. Moreover, only crude (non-age-adjusted) prevalence estimates were available from the CDC PLACES dataset, limiting the ability to account for differences in age structure across ZCTAs. Since age ≥65 was excluded in the final model, residual confounding from age distribution may still influence the observed associations, particularly if age is unevenly distributed across neighborhoods. Finally, selection bias may be present, as 32 ZCTAs were excluded due to incomplete overlap between ACS and CDC PLACES data, and these areas may differ systematically (e.g., by population size or geographic characteristics), potentially limiting generalizability. Additionally, unmeasured neighborhood-level factors, such as built environment, food access, and healthcare accessibility, may continue to influence spatial patterning. Despite these limitations, this study provides hypothesis-generating insights that may inform future surveillance and public health research directions in this medically underserved region.

## Conclusions

In summary, Riverside and San Bernardino Counties, as represented by the 132 ZCTAs measured in this study, have shown significant variation in hypertension and high cholesterol prevalence, alongside geographic clustering at the neighborhood-level. Furthermore, the application of spatially adjusted models indicated that obesity prevalence, median household income, and recent engagement with preventive care services were associated with cardiovascular risk; however, these findings may reflect differences in diagnosis and healthcare utilization rather than true disease burden. Interpreted within the context of increasing CVD hospitalization and mortality, these findings support the continued use of small-area spatial surveillance and may help inform future research evaluating place-based public health strategies.
